# Cellular Senescence as a Brake or Accelerator for Oncogenic Transformation and Role in Lymphatic Metastasis

**DOI:** 10.3390/ijms24032877

**Published:** 2023-02-02

**Authors:** Priyanka Banerjee, Niyanshi Gaddam, Tej K. Pandita, Sanjukta Chakraborty

**Affiliations:** 1 Department of Medical Physiology, Texas A&M Health Science Center, Bryan, TX 77807, USA; 2 Center for Genomics and Precision Medicine, Texas A&M College of Medicine, Houston, TX 77030, USA

**Keywords:** senescence, senescence-associated secretory phenotype (SASP), lymphatics, lymphangiogenesis cancer

## Abstract

Cellular senescence—the irreversible cell cycle arrest driven by a variety of mechanisms and, more specifically, the senescence-associated secretory phenotype (SASP)—is an important area of research in the context of different age-related diseases, such as cardiovascular disease and cancer. SASP factors play both beneficial and detrimental roles in age-related disease progression depending on the source of the SASPs, the target cells, and the microenvironment. The impact of senescence and the SASP on different cell types, the immune system, and the vascular system has been widely discussed. However, the impact of replicative or stress-induced senescence on lymphatic biology and pathological lymphangiogenesis remains underexplored. The lymphatic system plays a crucial role in the maintenance of body fluid homeostasis and immune surveillance. The perturbation of lymphatic function can hamper normal physiological function. Natural aging or stress-induced premature aging influences the lymphatic vessel structure and function, which significantly affect the role of lymphatics in tumor dissemination and metastasis. In this review, we focus on the role of senescence on lymphatic pathobiology, its impact on cancer, and potential therapeutic interventions to manipulate the aged or senescent lymphatic system for disease management.

## 1. Introduction

In the 21st century, with the advancement of science, improvement in quality of life, and scholars winning the battle against many diseases, people now have much longer life spans. According to World Population Prospects 2019 (United Nations, 2019), in 2019, 1 in 11 people in the world were >65 years old, and that number will become 1 in 6 people by 2050 (United Nations, Department of Economic and Social Affairs, Population Division (2019) (World Population Ageing 2019: Highlights (ST/ESA/SER.A/430)). Aging and age-related diseases such as malignant neoplasm, heart diseases, Alzheimer’s disease, and diabetes mellitus are the leading cause of death in humans [1]. In the recent COVID-19 pandemic, advanced age (>65 years) emerged as one of the major risk factors for fatality [2]. Although natural aging is inevitable, it is necessary to delineate the molecular signatures associated with aging and age-related diseases to manipulate premature aging and prevent those diseases. Cellular senescence is one of the key factors of aging [3,4]. The role of cellular senescence on cardiovascular diseases, cancer, vascular endothelial cells, and immune cells has been broadly discussed [5,6,7], but the effects of senescence on the lymphatic system and its impact on age-related diseases overall warrant further evaluation. In this review, we will discuss cellular senescence in the context of aging or age-related diseases, and its impact on the lymphatic vascular system.

## 2. Senescence and Senescence-Associated Secretory Phenotype (SASP)

### 2.1. Senescence

Cellular senescence is defined as a stress-responsive stable cell cycle arrest [1,2], which has been associated with shortened telomeres [3]. At senescence, cells are metabolically active and viable but no longer divide. This was first characterized by Hayflick and Moorhead [4]. Senescent cells are distinguished by their dynamic pathophysiology resulting from their departure from the cell cycle and their morphological and metabolic reconfiguration that enable them to differentially contribute to various pathological conditions, aging, and tissue remodeling [5]. As telomeres intrinsically shorten and modify conformations through the natural passage of time, replicative senescence (RS) culminates through the DNA damage response (DDR) to exposed chromosomal ends. This occurrence is widely thought to be a driving factor in aging, with the deleterious accumulation of senescent cells in age-related pathologies remaining unchecked by evolutionary processes of selection [6]. In addition to RS, senescence can also be induced by internal and external stimuli such as genotoxic agents, stress, mitochondrial dysfunction, oncogene activation, and chemotherapy, and is referred to as stress-induced premature senescence (SIPS) [7].

Senescent cells secrete a cocktail of proinflammatory cytokines, chemokines, growth factors, proangiogenic factors, reactive oxygen species (ROS), and proteases that represent the senescence-associated secretory phenotype (SASP) [7]. Senescent cells further communicate with neighboring cells and the immune system via the SASP in an autocrine or paracrine manner. The cytokines and chemokines secreted by senescent cells recruit T-cells, macrophages, and natural killer cells that, in turn, aid the removal of the senescent cells [7] to maintain tissue homeostasis. With the increase in age, the weakened immune system, or manifestation of ‘immunosenescence’, fails to clear the senescent cells, which results in their accumulation over time. In addition to the crosstalk with the surrounding immune cells, senescent cells also further induce senescence in neighboring cells by the secretion of SASP factors and extracellular vesicles (EVs).

#### Cellular Senescence: Double Edged Sword for Cancer

The role of the SASP on the surrounding cells in cancer progression or cancer prevention is very much context dependent. The SASP results in the secretion of numerous proinflammatory cytokines and chemokines promoting the dedifferentiation and proliferation of neighboring metastatic cells. These chemokines and cytokines in turn attract immune cells to the tumor site and help with the immune clearance of the malignant cells [8]. On the other hand, the SASP can serve as an inducer of tumorigenesis. In an in vitro model of ovarian cancer, it has been shown that when non-neoplastic cells were treated with the conditioned media (CM) from senescent fibroblasts, neoplastic transformation was induced in those cells [9]. Interleukin-6 (IL-6) and Interleukin-8 (IL-8) are some of the common SASP factors inducing tumorigenesis in breast, prostate, and lung cancers [10,11,12,13]. Further, senescent cells share some common characteristics of cancer-associated fibroblasts (CAFs) [14,15,16]. Further, senescent cells also promote tumorigenesis via the production of matrix metalloproteinases (MMPs) as SASP factors that enable the restructuring of the extracellular matrix (ECM) and facilitate tumor growth [17,18,19]. Senescent cells, via SASP factors, can induce the epithelial-to-mesenchymal transition (EMT) [8,20] and also promote an immunosuppressive tumorigenic microenvironment. Senescent hepatocytes in hepatocellular carcinoma attract immunosuppressive Cd11b^+^Gr1 myeloid cells that inhibit T-cell proliferation and contribute to tumor progression [21]. Additionally, the SASP can also induce chemoresistance in cancer cells. When malignant pleural mesothelioma (MPM) cells that show significant chemoresistance were treated with pemetrexed for 96 h in vitro, 60% of the cells became senescent. The conditioned media (CM) from the pemetrexed-treated senescent MPM cells induced EMT with an increased expression of vimentin, fibronectin, slug, and snail in previously nonsenescent cells [20]. In the process of cancer progression and metastasis, senescent cells also increased the angiogenesis by producing angiogenic factors, such as vascular endothelial growth factor (VEGF) and connective tissue growth factor (CTGF) [22,23,24].

**Figure 1:**
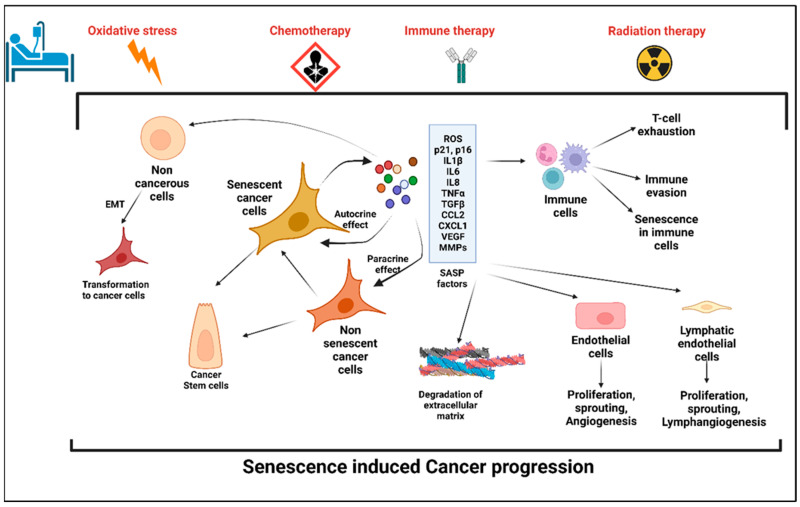
Stress-induced senescence, SASP, and cancer progression. Anticancer therapy including chemotherapy, radiation therapy, and immune therapy induced premature senescence in cancer cells and the cells in the tumor microenvironment (TME). Senescent cancer cells secrete plethora of proinflammatory cytokines, chemokines, matrix metalloproteinases, angiogenic factors, and reactive oxygen species, which are collectively known as senescence-associated secretory phenotype (SASP). SASP factors induce senescence in the cells in the TME, which in turn evade the immune response. Factors secreted by senescent cells promote angiogenesis and lymphangiogenesis by its effects on neighboring endothelial cells and hence enhances cancer cell metastasis. SASP-induced cancer stemness increases the proliferation and self-renewal properties of the cancer cells.

Interestingly, senescent cells also acquire a higher potential of invasiveness and lymphangiogenesis. In several metastatic cancers, lymph node metastasis and increased lymphangiogenesis are one of the prognostic factors [25,26,27]. Kim et al. showed that there was an accumulation of senescent cells in the front region of a collective invasion of papillary thyroid carcinoma (PTC), and also within the lymphatic vessels and the metastatic lymph node, which indicate their role in tumor progression and LN metastasis [28]. Further, it also lends credence to the hypothesis that metastatic tumor cells within the lymph nodes acquire vulnerabilities that help to evade traditional therapies and become more aggressive. Importantly, a new role for senescent cells in tumorigenesis has recently emerged, demonstrating that therapy-induced senescent cells can acquire stemness (SAS: senescence-associated stemness) [2,29], and that acquired stemness assists senescent cells in escaping from cell cycle arrest and harnessing an aggressive growth potential (Figure 1).

### 2.2. Anticancer Treatment: A Potential Trigger to Cellular Senescence

The role of conventional anticancer therapies in the induction of cellular senescence in cancerous or noncancerous tissues is an important area of research. The use of chemotherapies, radiation therapy, and also immunotherapy significantly induce senescence.

**A.**  **Chemotherapy and senescence**

The general mode of action of chemotherapy is to impair the mitosis in cancer cells and disrupt the DDR [30]. Doxorubicin, Etoposide, and Camptothecin are common topoisomerase inhibitors that inhibit the progression of replication and are used as chemotherapeutic agents for colon cancers, breast cancer, hepatocellular carcinoma, lung cancers, and acute lymphocytic leukemia [30,31,32,33]. A recent study by Karabicici et al. showed that Doxorubicin treatment induced senescence in both liver cancer stem cells (EpCAM+/CD133+) as well as in a non-stem-cell population (EpCAM-/CD133-nonstem) in the Huh7 cell line with a concomitant increase in the reprogramming genes (SOX2, KLF4, c-MYC), liver stemness-related genes, (EpCAM, CK19), and ANXA3 in those cell populations [34]. The conditioned media from the Doxorubicin-treated cells contained high levels of inflammatory cytokines, IL8, and Interferon Gamma Induced Protein-10 (IP10) [34]. Doxorubicin treatment also caused cardiotoxicity in cancer survivors by inducing cardiomyocyte senescence [35]. Along with the topoisomerase inhibitors, alkylating agents are another group of chemotherapies used for several cancers as they inhibit DNA replication [35]. Two commonly used alkylating agents for cancer treatment are Cisplatin and Temozolomide. Temozolomide is the first-line therapeutic for high-grade glioblastoma. A recent study showed that temozolomide induced cellular senescence in the glioblastoma cells at a four-fold higher level than cellular apoptosis. Interestingly, compared to the primary tumors, the population of senescent cells was significantly higher in the recurrent cancer tissues. The high population of senescent cells upon temozolomide treatment contained elevated levels of proinflammatory cytokines including IL-1α, IL-1β, IL-6, and IL-8, as well as CCL2, CCL8, and CXCL1. The proinflammatory cytokine cocktail present in the tumor microenvironment of temozolomide glioblastoma potentially accelerated tumor growth and relapse [36]. Cisplatin-induced cellular senescence in cancer has been well reported in several articles [37,38,39,40]. Microtubule inhibitors such as Paclitaxel arrest the cells at mitosis by interfering with microtubule dynamics [41]; however, like other chemotherapeutic agents, microtubule inhibitors have been reported to induce cellular senescence in cancer as well as noncancer cells [42]. Table 1 summarizes the role of chemotherapies on the induction of cellular senescence in cancer patients.

**B.**  **Radiation-induced senescence**

Radiation therapy (RT) is used to kill the cancer cells by inducing irreparable DNA damages in a nonspecific manner. Ionizing radiation (IR) is one of the potent RTs for cancer patients with a wide range of cancers, including lymphoma, soft tissue sarcoma, head–neck cancer, breast cancer, and lung cancer [60]. Depending on the dose and fraction of the IR regimen, cancer cells exposed to IR are arrested at different stages of the cell cycle, i.e., in the G1, G2, or S phase [61]. The dose of IR also determines the induction of cellular senescence or apoptosis. Studies have shown that a high dose of IR (>10 Gy) to endothelial cells (ECs) induced apoptosis while a moderate dose (>0.5 Gy) of IR induced senescence [62]. As listed in Table 1, IR can induce senescence with the overexpression of p16, p21, and beta-galactosidase activity in the exposed cells.

**C.**  **Immunotherapy-induced senescence**

Immunotherapy is currently a promising anticancer therapy for several cancers. For antitumor immunity, both the Th1 and Th2 CD4^+^-T-helper cells play a crucial role by inducing cellular and humoral immunity, respectively [63,64]. A study of the carcinogenesis in pancreatic islets showed that T-antigen-specific CD4^+^ Th cells induced growth arrest of proliferating tumor cells without any significant cytotoxic effects [65]. That study also highlighted the possibility of a noncytotoxic way to induce cellular growth arrest or cellular senescence mediated by Th1, Th2 cytokine immunotherapy [65]. In invasive β-cell cancers, Interferon-gamma (IFN-ϒ)- and Tumor Necrosis Factor (TNF)-producing CD4^+^ Th1 cells induce senescence in β-cells via the STAT1- and TNFR1-dependent stabilization of the p16INK4a–Rb [66]. In triple negative and HER2^+^ breast cancer cells, treatment with either CD4^+^ Th1 cells or Th1 cytokines TNF-α and IFN-γ induced apoptosis and tumor senescence [67]. In B cell lymphoma, CD20-targeted immunotherapy induced senescence in the cancer cells by enhancing the levels of cellular reactive oxygen species (ROS), which is an important SASP factor and also sensitizes the cells to the DDR [58]. The senescent cancer cells can attract the other immune cells in the primary tumor site and help with the immune-mediated clearance of cancer cells via the SASP (Figure 1).

### 2.3. The Cellular and Molecular Mechanism of SIPS

#### 2.3.1. Mitochondrial Dysfunction and SIPS

Mitochondrial dysfunction contributes to premature senescence [68]. One of the potential mechanisms of dysfunctional mitochondria-induced senescence is excessive ROS production. Excessive ROS can lead to DNA damage and induce senescence [69]. A recent study by Kotla et al. showed that cancer treatment with IR or Doxorubicin (i) increased mitochondrial ROS (mtROS) production and (ii) caused mitochondrial stunning (the reversible mt dysfunction), (iii) which then activated the p90RSK/ERK5-S496 complex and decreased the nuclear factor erythroid 2-related factor 2 (NRF2) transcriptional activity, and (iv) the reduced NRF2 transcriptional activity reduced the expression of antioxidant genes (HO1 and Trx1). ROS further cause telomeric DNA damages in the nucleus via poly (ADP-ribose) polymerase (PARP) activation and consequently deplete the NAD+ level and lead to further mtROS production. Altogether, a positive feedback loop is established between the nucleus and mitochondria, which reprograms the neighboring myeloid cells to induce a sustained SASP state [70].

#### 2.3.2. Molecular Pathways

**A.**  **Cyclic GMP–AMP synthase (cGAS)–stimulator of interferon genes (STING) (cGAS-STING) pathway**

**Figure 2:**
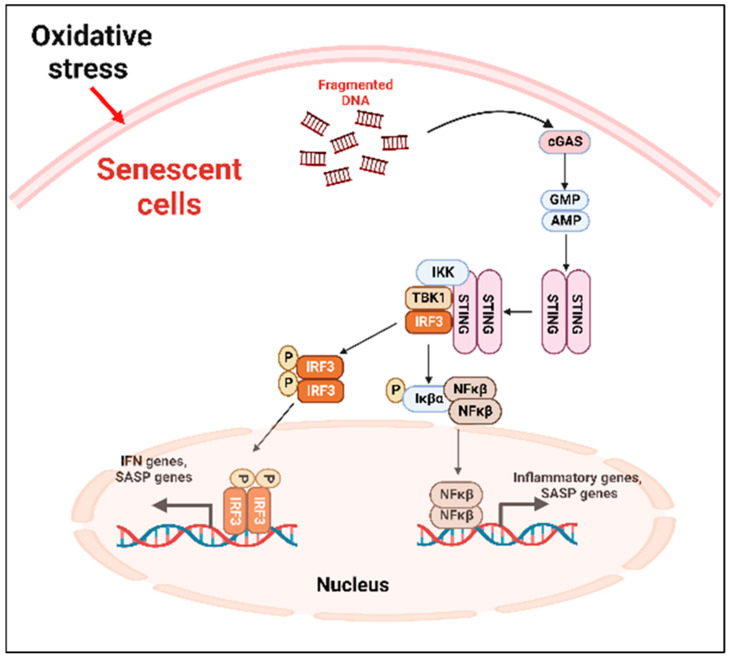
cGAS-STING-mediated cellular senescence. In response to oxidative stress, DNA fragments are released from the nuclei to the cytoplasm which are recognized by the cGAS, activating STING. The activated STING complex with TBK1 phosphorylates the transcription factor IRF3 and NFκβ. Activated NFκβ induces the transcription of proinflammatory SASP genes. cGAS: cyclic guanosine monophosphate (GMP)-adenosine monophosphate (AMP) synthase; STING: stimulator of interferon genes; TBK1: TANK-binding kinase 1; NFκβ: nuclear factor ‘kappa-light-chain-enhancer.

SASP factors can be expressed by various mechanism depending on the cell types [71]. The nuclei of the primary senescent cells released fragmented genomic DNA into the cytoplasm. The cyclic guanosine monophosphate (GMP)–adenosine monophosphate (AMP) synthase (cGAS) senses the cytoplasmic DNA in the form of cyclic dinucleotides. The cyclic GMP-AMP (cGAMP) complex then activates a stimulator of interferon genes (STING) located in the endoplasmic reticulum. The activated STING combines with TANK-binding kinase 1 (TBK1) and phosphorylates the transcription factors interferon regulatory factor 3 (IRF3) and nuclear factor ‘kappa-light-chain-enhancer’ (NFκB), causing its nuclear translocation and the further activation of the SASP gene expression (Figure 2) [71].

**B.**  **p53 pathway**

**Figure 3:**
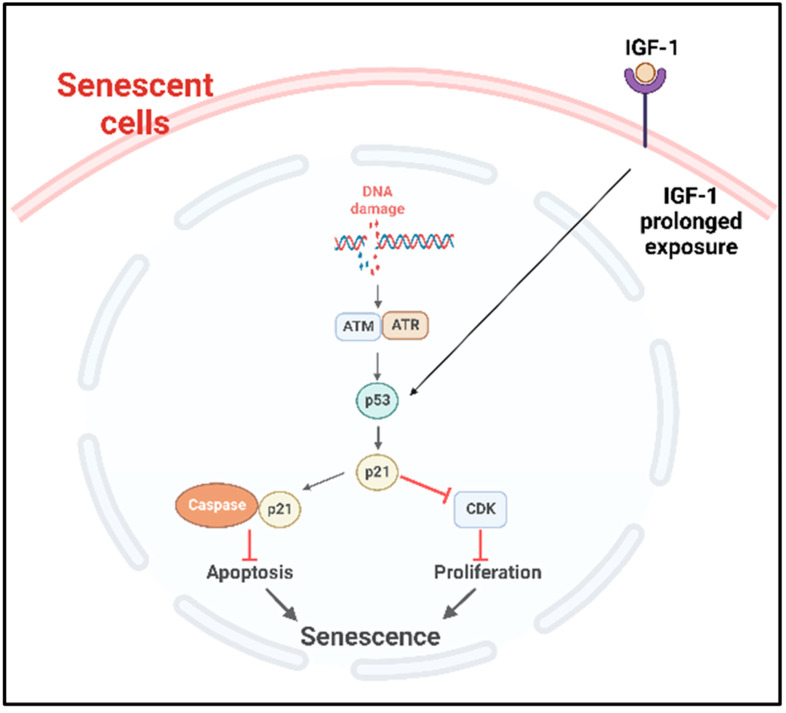
p53 mediated cellular senescence. In response to DNA damage caused by DNA damaging factors, the DDR pathway is activated with the concomitant activation of ATR or ATM which in turn stabilize p53. Prolonged exposure of the cancer cells to IGF-1 also causes the stabilization and activation of p53 pathway. Consequently, the downstream p53/p21cip1 are activated, which can then inhibit the apoptosis by binding with apoptotic genes including caspases and induce senescence. Activated p21^cip1^, which is a member of the CDK inhibitor, inhibits the cellular proliferation. DDR: DNA damage response, ATM: ataxia telangiectasia mutated, ATR: ataxia telangiectasia and Rad3-related kinase, IGF-1: insulin-like growth factor-1, CDK: cyclin-dependent kinases.

p53 plays an important role in the onset of cellular senescence. The telomeric erosion and the DNA damage response pathway leads to the activation of p53 [72,73]. In response to the DDR, the stress sensors’ telangiectasia-muted (ATM) or ataxia telangiectasia and Rad3-related (ATR) kinases are activated, which in turn activate the p53/p21^cip1^ with p53 stabilization [72]. The p21^cip1^ is one of the founding members of the mammalian CDK inhibitor family and, upon activation, p21^cip1^ binds with many apoptotic genes, including caspases; as a result, it inhibits apoptosis and induces senescence [74]. p53 also plays as a molecular switch in the insulin-like growth factor-1 (IGF-1)-induced cellular premature senescence. Increased IGF-1 levels are associated with cancer progression [75]. Tran et al. have shown that long-term IGF-1 exposure increased p53 acetylation, leading to p53 stabilization which ultimately induced premature senescence (Figure 3) [76].

**C.**  **NFκβ pathway**

**Figure 4:**
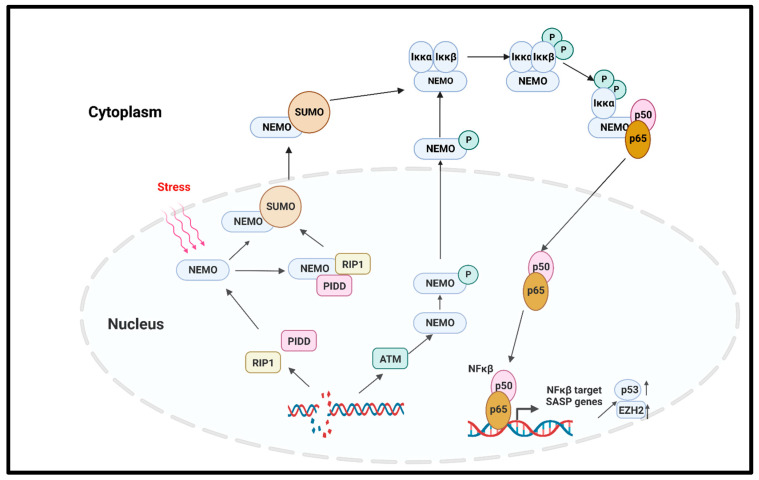
NFκβ-mediated cellular senescence. Extracellular stress or DNA damage caused by extra- or intracellular factors activate the NEMO, which is the regulatory subunit of IKK complex. In response to genotoxic stress, the p53-induced protein with death domain (PIDD) and receptor-interacting protein kinase 1 (RIP1) SUMOylate NEMO, and SUMOylated NEMO is exported to the cytoplasm. The DNA-damage-induced activation of ATM also phosphorylates the NEMO and causes its nuclear export. In the cytoplasm, NEMO activates IKK complex, phosphorylates the Iκβα, and induces its proteasomal degradation. p65/p50 heterodimer is released and transported to the nucleus to activate the NFκβ signaling, and as the downstream effect, SASP genes are expressed. NEMO: NFκβ essential modulator; IKK: Iκβ kinase; PIDD: p53-induced protein with death domain; RIP1: receptor interacting protein kinase 1.

In solid tumors, the noncanonical NFκβ pathway activation leads to senescence via the regulation of the enhancer of Zeste homologue 2 (EZH2) [77]. EZH2 is significantly increased in hematopoietic and solid tumors [78]. The overexpression of EZH2 suppresses the senescence by inhibiting p21^Cip1^ (CDKN1A) in a p53-independent manner [79]. Another important regulator of the canonical NFκβ pathway that induced premature senescence in cancer is the DNA damage which activates the NFκβ pathway with NFκβ essential modulator (NEMO) protein. NEMO, the regulatory subunit of the Iκβ Kinase Complex (IKK) complex protein, regulates NFκβ signaling via the regulation of the IKK complex [80]. The genotoxic stress-induced SUMOylation of the NFκβ essential modulator (NEMO) can also be promoted by p53-induced protein with a death domain (PIDD) and receptor-interacting protein kinase 1 (RIP1). The SUMOylation of NEMO induces its nuclear export. Additionally, the stress-induced double-stranded break (DSB) of DNA activates the ATM, which in turn phosphorylates NEMO, inducing its monoubiquitination and nuclear export. As a consequence, the IKK complex is activated, and the activated IKK phosphorylates Iκβα and its proteasomal degradation. Finally, the p65/p50 heterodimer is released and translocated to the nucleus to activate the NFκβ signaling cascade [81,82]. A study by Dong et al. reported that radiation-induced endothelial cell senescence is caused by the activation of the DSB/NEMO/NFκβ signal pathway (Figure 4) [83].

An essential regulator of the TNF-induced NFkβ pathway is ataxia-telangiectasia mutated (ATM), a master regulator of the DNA double-strand break (DSB) repair pathway after genotoxic stress [84]. The downstream target of damage ATM for the cell cycle checkpoint is p53, which is regulated by ATM-dependent phosphorylation [85]. Cells defective in ATM function have defective telomere metabolism [86,87,88] as well as a higher frequency of SA-β-gal [89], which are both phenotypes associated with senescence. The agents causing DSBs lead to p16INK4a enrichment and the premature senescence of normal fibroblasts [90]. A transient increase in p21 is followed by a delayed induction of p16INK4a, which also happens with the permanent arrest that is observed with cellular senescence. These observations have indicated that damage-induced cells are very similar to senescent cells and have additional factor(s) beside p21 and p53 that maintain cell cycle arrest [90].

**D.**  **Mammalian target of rapamycin (mTOR) pathway**

mTOR is the intracellular target of the pharmacological drug rapamycin, which is widely used in many cancers. The mTOR pathway positively regulates the protein synthesis pathway and inhibits autophagy, thereby playing an important role in senescence [91,92]. As discussed earlier, cellular senescence is associated with mitochondrial dysfunction, resulting in impaired ATP generation and increased reactive oxygen species (ROS). Mitochondrial metabolism and biogenesis are regulated by the master regulators Peroxisome proliferator-activated receptor gamma (PPARγ) and its coactivator 1(Peroxisome proliferator-activated receptor-γ coactivator, PGC1-α). Importantly, mTORC1, one of the TOR complexes (mTROC1 and mTORC2), regulates the transcriptional activity of PGC1-α [93]. Thus, rapamycin treatment to inhibit mTOR signaling reduces radiation-induced ROS production, inhibits senescence, and increases cellular life span [94,95].

**E.**  **Transforming growth factor-β (TGFβ) pathway**

The role of TGFβ in premature senescence has been reported in several studies in multiple cell types, which include bronchial epithelial cells and hepatocellular carcinoma cells [96,97,98]. TGFβ induced the cyclin-dependent kinase inhibitors p15Ink4b, p21, and p27 and suppressed cellular proliferation [99,100]. TGFβ was also reported to induce ROS production in mitochondria in different cell types [101,102], and ROS are one of the inducers of premature senescence. Another TGFβ-targeted gene which is an important regulator of senescence and age-related diseases is plasminogen activator inhibitor-1 (PAI-1) [103]. Interestingly, TGFβ is also considered an important SASP factor, and it causes senescence in cells by autocrine and paracrine manners. In senescence, the polycomb protein Chromobox 7, CBX7, affects the upregulation of integrin β3 (ITGB3), which in turn activates the TGFβ signaling in an autocrine and paracrine manner in human fibroblast [104].

**F.**  **Mitogen-activated protein kinase (MAPK) pathway**

Genotoxic stress in senescence activates the p38 MAPK pathway, which is independent of the DDR [105]. In senescent cells, p38 MAPK regulates the NFκβ activity; the role of NFκβ in senescence was discussed in a previous section [105]. In senescent T-cells, the intracellular metabolic sensor AMPK activates p38 via its autophosphorylation via the scaffold protein TAB1 [106]. The activation of this pathway leads to the inhibition of telomerase activity, T-cell proliferation, and senescence [106].

### 2.4. Telomerase Activity Suppresses Senescence and Its Inhibition Enhances Senescence

Telomerase consists of an RNA component (hTR), which serves as a telomeric template and a catalytic protein component (hTERT), which has a reverse transcriptase activity [107,108,109]. The ectopic expression of hTERT prevents replicative senescence in several cell types, including fibroblasts and epithelial cells, by exerting antiapoptotic action in early stages of the cell death prior to caspase activation and mitochondrial dysfunction [110,111]. Immortalization in human cells has been achieved by the expression of hTERT [89], which results in the loss of p16-dependent cell cycle control [112,113]. The inhibition of telomerase activity via treatment with GRN163L (human telomerase RNA-targeted antisense agents) inhibits cell growth [113], supporting the argument that telomerase regulates senescence.

## 3. Lymphatic System: A Critical Regulator of Fluid Homeostasis and Immune Response

The lymphatic vascular system is crucial for maintaining body fluid homeostasis; the transportation of excess interstitial fluid, antigens, and activated immune cells during inflammation; and facilitating macromolecule absorption [114,115,116]. The lymphatic system is reported to play vital roles in almost all organs of the body [117].

### 3.1. Structural Components of Lymphatic System and Its Function

The lymphatic vascular system comprises blind-ended capillaries, precollecting and collecting vessels and draining lymph nodes (LN) [118]. The internal wall of the lymphatic vasculature is layered with lymphatic endothelial cells (LECs). The LECs have unique features with a different transcriptional profile, which make them distinct from the blood endothelial cells (BECs). LECs have a high level of expression for the transcription factor, Prospero homeobox protein 1, PROX1, and also the factors such as O-Glycoprotein Podoplanin, Lymphatic vessel endothelial hyaluronan receptor-1 (LYVE-1), vascular endothelial growth factor receptor-3 (VEGFR3), and neuropilin-2 (Nrp-2) [119,120].

The first entry of fluid into the lymphatics is driven by hydrostatic and osmotic pressure gradients [121]. The blind-ended, highly permeable lymphatic capillaries have discontinuous button-like junctions on the membrane structure, a single layer of LECs, and lack the pericytes and smooth muscle cells (SMC) [121,122]. The lymphatic capillaries collect the interstitial fluid (IF) from the nearby blood capillaries and, through the precollecting vessels, transport IF to the collecting vessels. Along with IF, immune cells enter the lymphatics, although the entry is cell specific. In general, dendritic cells (DCs), macrophages, and lymphocytes, but not the neutrophils and erythrocytes, enter the lymphatics [121]. The chemokines and cytokines from the LECs attract the leukocytes towards the lymphatics [118,121]. In contrast with the initial lymphatic capillaries, the larger secondary collecting lymphatics are lined with LECs tightly connected with each other, and these vessels are also covered with specialized contractile lymphatic muscle cells [118,122] and contain valves that open and close depending on the sequential fluid pressure [118]. The lymphatic fluid then gets transported to the lymph node (LN) through the afferent collecting vessels, and then through a sequence of nodes via efferent collecting vessels [118]. The LNs help with the expansion of the immune response and also serve as a barrier by preventing the harmful stimuli from returning to the blood circulation [122].

### 3.2. Aging and Effects on Lymphatic Function and Pathophysiology

#### 3.2.1. Lymphatic Inflammation and Lymphangiogenesis

The lymphatic vasculature, composed of endothelial cells interlinked by vascular endothelial-cadherins on the ECM, is susceptible to hyperpermeability upon inflammation. Inducers of inflammation include histamine, thrombin, vascular endothelial growth factor (VEGF), IL-1, IL-6, and TNF-α [123], and all are noted to increasingly circulate with the progression of age [124]. These immune cells and proinflammatory cytokines propagate intercellular signaling, promoting wider endothelial gaps and the loss of junction integrity. This pattern enables the broadened lymphatic uptake of infiltrating cancer cells and promotes the establishment of a prometastatic niche and subsequent cancer dissemination. Lymphangitis additionally expresses VEGF-A and VEGF-C, consequently activating VEGFR2, a known causal factor for lymphangiogenesis [125]. Amplifying the lymphatic vasculature provides a vaster surface area for cancer cell uptake, often working in conjugation with lymphangitis to permit metastasis.

The correlation to age denotes the prevalence of cellular senescence in both lymphangitis and lymphangiogenesis, with many of the same proinflammatory agents (IL-1β, IL-6) being established SASP factors [126]. Lymph node metastasis is highly prognostic to cancer development, with nodal status often determining subsequent treatment and survivability [127,128]. Malignant cancers such as lymphoma, leukemia, and metastatic cancers generate lymph node swelling—referred to as lymphadenopathy—that worsens the condition of the present diagnosis as tumor cells flourish within the immunosuppressive environment created [129]. The poor prognosis is exacerbated with age, attributed to the minimized clearance of tumor cells at the primary and metastatic sites. More research observing and treating lymph node infiltration and inflammation should be conducted to better determine these mechanisms.

#### 3.2.2. Lymphatic Contractility

The pumping mechanism of the lymphatic system is integral to maintaining fluid homeostasis, cellular waste removal, lipid absorption, and lymphocyte production, and the impairment of this function has significant pathophysiological implications [130]. A pressure gradient governs the lymphatic contractions, with both intrinsic and extrinsic pumps responding to lymph pressure changes and guiding the flow unidirectionally [131]. VEGF-C and VEGF-D, secreted by tumors, induce a greater contraction of proximal lymphatic vessels through lymphangiogenesis and are adept disseminators for nearby tumor cells [132]. These routes expand and evade the growing tumors, allowing the cancer to further metastasize throughout the extensive transit network generated.

In contrast to the tumor-proximate lymphatics that increase the contraction rates of lymph flow, an evident decrease in lymphatic contractility has been observed in aged lymphatics [133]. A significant decrease in the lymphatic vessel contractile function, lymph pump, and fractional flow are observed due to fundamental alterations to the lymph pump function with progressive aging [134]. Further, prevalve and valve zones demonstrate significant aging-associated decreases in muscle cells that could potentially have a direct impact on the vessel biomechanics and limit the response of the lymphatic vessels to clear inflammation in the elderly, perpetuating the progression of inflammatory damage [135]. Rather than contradicting the previous finding, however, it is likely the unison of the two that bolsters cancer progression. The lowered contractility in elderly patients culminates from decreasingly efficient lymph pumps [136] and the depletion of contractile proteins such as troponins, myosin, and other cytoskeleton-associated proteins [133]. This degeneration of the pumping mechanism reduces the antigen movement and cancer cell clearance that would mitigate the problem, granting lymph nodes invaded by tumor cells the chance to proliferate to malignance. The combination of increased lymph flow proximal to the primary tumor sites and lack of clearance of the sentinel and regional lymph nodes overall benefits metastasis, an occurrence bolstered through age and lymphatic modifications, which was explained in previous sections.

#### 3.2.3. Immunosuppression of Lymph Nodes in Tumor Microenvironment

Significant alterations in mast cell function have been shown to be associated with increased inflammatory microenvironment as well as the impaired function of lymphatic vessels with the onset of aging [137]. Lymph nodes are the sites for the activation and maturation of lymphocytes, where they encounter the free form of antigens, or the antigens presented by the antigen-presenting cells such as DCs. The naïve T-cells enter the LN through either the high endothelial venules (HEVs) or afferent lymphatic vessels [138,139] and then are compartmentalized into different subcompartments in response to the gradient of chemokines, e.g., CCL19 and CCL21, and contact with the antigen-presenting DCs. Upon introduction to the specific antigens, T-cells get activated, which is crucial for the adaptive T-cell-mediated immune response [140]. Activated T-cells express the C-X-C chemokine receptor type 5 (CXCR5), which acts as the predominant helper cells for B cell activation. The CXCR5^+^CD8^+^ cells interact with CXCL13 expressing B cells in the follicle [141]. Those CXCR5^+^CD8^+^ cells have a low level of expression of immune evasion molecules, such as PD-1 and TIM-3, and a high level of proinflammatory cytokines such as TNF-α and IFN-γ [142]. In senescent conditions, the number of naïve T-cells diminish and the population of memory T-cells increase, which inhibits the immune cell response to new antigens [139]. A recent study by Ramello et al. reported that in breast cancer patients, there was an infiltration of senescent T-cells in the tumor-draining lymph nodes, and the further characterization of those senescent T-cells revealed a high enrichment of T-cell exhaustion markers [143]. Not only the T-cells, but also the B-cell populations, maturation, and proper compartmentalization are disrupted with age. A study on nonhuman primates showed that, with aging, the number of proliferating (Ki67^hi^) B cells in the germinal center of the lymph node decreased with a concomitant increase in the suppressor FoxP3^hi^ Lag3^hi^ CD4 T-cells [144].

As tumor cells continue to invade lymph nodes, an immunosuppressive environment is induced that further promotes tumor growth. Senescent cells accumulate proximal to the tumor, and SASP signal pathways and transcription networks likely play an important role. While prosenescence treatments beneficially terminate the replication of cancerous cells, these now senescent cells withhold autocrine and paracrine signaling with the potential to again propagate tumor relapse in the future [145]. Common SASP factors, such as IL-6 stromal cells, have displayed immunosuppressive qualities through paracrine signaling. In this study, senescent cells in the stroma limited the T-cell immune response to MK16-Ras and PDSC5 tumor cells and promoted growth [146]. CCL2, another established SASP factor, has additionally showcased a recruiting capability of immunosuppressive myeloid cells and encourage the growth of hepatocellular carcinomas [21].

**Figure 5:**
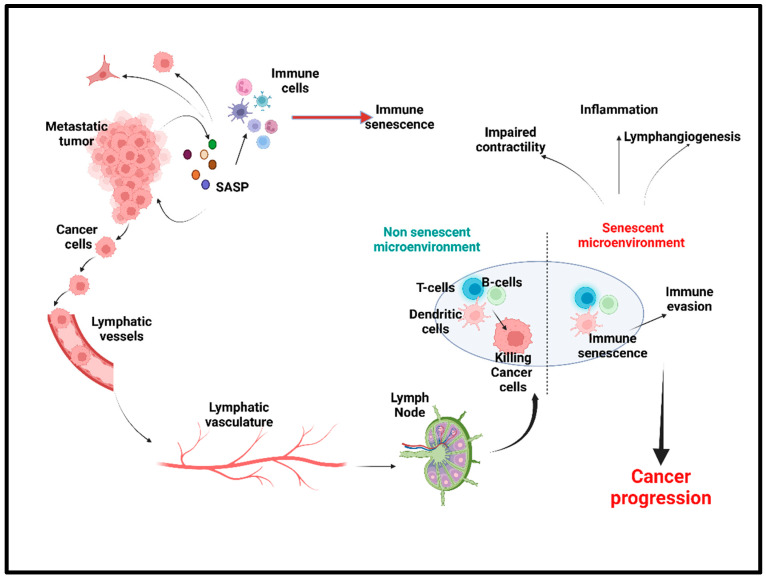
Immunosenescence in cancer microenvironment and immune evasion in lymph node Senescence-induced immunosuppression causes the immune evasion of cancer cells in lymphatic system, especially in tumor-draining lymph node (LN). In healthy, young lymph nodes, naïve T-cells enter the LN, compartmentalize, and mature. Upon interaction with the antigen-presenting cells, DCs, the T-cells get activated and help with the B-cell mediated immune response and help with tumor-cell killing. In the aged LN, or prematurely senescent LN, the T-cells become senescent and express the immune evasion markers including PD-1 and TIM3 and the compartmentalization and maturation of B-cells are disrupted. Consequently, the T-mediated tumor-cells killing is disrupted. The senescent lymphatic system is presented with impaired contractility and enhanced inflammation, which also increased the lymphangiogenesis in the tumor-bearing beds. LN: lymph nodes, DC: dendritic cells.

In a protumorigenic location such as the LN, the decreased ability to recruit immune cells because of senescence directly correlates with increased tumor development. Senescent cells are shown to actively secrete immunosuppressive cargoes that further increase the senescence of recipient immune cells such as M2 macrophages, myeloid-derived suppressor cells, and regulatory T-cells that further contribute to the inflammatory cascade and accelerate senescence and tissue aging [147]. It has additionally been demonstrated that dendritic cell recruitment in mouse lungs was decreased by the age-related increase in prostaglandin-2, diminishing the T-cell response [148]. Altogether, these findings showcase the competence of senescent cells for clearing way to tumor cells within the constructed immunosuppressive environment (Figure 5).

## 4. Prosenescence Mechanisms in Different Cancer Treatments

Many cancer treatments are intentionally imbued with prosenescence functionality to mitigate cancerous cell replication; however, the SASP-associated pathways can become detrimental and must be considered before the induction of senescence. The chemotherapeutic drug cyclophosphamide, for example, invokes senescence through the upregulation of the p53 and p16 genes most often when apoptotic blockers deem it the sole route for cell cycle arrest [149]. These senescent cells, which offer the intended suppression of malignant cells early in treatment, continue to promote tumorigenesis in aged organisms, a finding showcased in malignant epithelial cells of mice xenografts [150]. Termed “antagonistic pleiotropy,” this juxtaposition encapsulates the senescent contradiction of the early gain and later cost to cancer patients undergoing prosenescence therapy.

The therapeutic promotion of senescence seen particularly in chemotherapies consequently enables the SASP alteration of tissue environments into protumorigenic sites. Inflammation induced by secreted SASP factors IL-6 and IL-8 contribute to this development, with chemotherapies such as such as Abemaciclib, Palbociclib, and Ribociclib supporting the senescent tumor cell proliferation and inflammatory environment that permits further invasion and metastasis [151]. Additional SASP factors derived from the senescent cells (i.e., IL-1α, CCL2, CXCL 1, and MMPs) further promote tumor growth through immunosuppression, greater cell aggressiveness, and expansion of vasculature proximal to tumorigenic sites. Of course, there is another side in which chemotherapies induce senescent cells with antitumor properties. Topetecan, for instance, was shown to recruit a favorable SASP for tumor regression when used on MYCN Proto-Oncogene (MYCN) neuroblastomas [152]. However, the same study denoted other prosenescent treatments such as bromo-deoxy-uridine as protumorigenic, exemplifying another instance of the high variability of senescent cells and the SASP when confronted with a cancerous environment.

## 5. Potential Senotherapies

The consequences inherent to senescent cell accumulation bolster the necessity for treatments that can mitigate their proliferation; hence, senotherapy is required. Depending on the mode of action, senotherapeutics are grouped into two classes, namely, senolytics and senomorphic drugs [153,154]. Senolytic compounds induce the senolysis of senescent cells, leading to the selective elimination of senescent cells. Senolytic drugs comprise those that target antiapoptotic proteins such as CL-2/BCL-XL family members to induce apoptosis, p53 inhibitors, or molecules targeting the NFκβ pathway, PI3K/AK pathways, etc. Senomorphic drugs attenuate the SASPs without inducing the apoptosis of senescent cells [153]. Senomorphic drugs include but are not limited to the inhibitors of Ikβ kinase (IKK) and nuclear factor (NF)-kβ, free radical scavengers, and rapamycin (the mTOR inhibitor), which have been discussed in several reviews [153,154,155]. The combination of two drugs, Dasatinib (D) and Quercetin (Q), were the first senolytic drugs reported, and later on, many other senolytic and senomorphic drugs were tested in vitro and in vivo (Table 2). It is important to mention that although the D+Q combination acts as senolytic, the Q has both senolytic and senomorphic properties because it can affect the pathways including p53/p21/serpines, PI3K/Akt/mTOR, and NF-κβ signaling [156,157,158]. The quercetin-related flavonoid Fisetin also showed both senolytic and senomorphic potential [153]. Since senolytic drugs target senescent cells, which are accumulated over the time, senolytic drugs are administered on a short-term basis. On the other hand, senomorphic drugs are given chronically for the sustained suppression of SASPs, thereby reducing the slowly growing population of senescent cells [153,154]. Many factors demand consideration when determining senolytic drug usage, with the crucial one being how to safely target senescent cells in a controlled manner. Inducing senescence, as seen in many cancer therapies, embodies benefits in the pursuit of inhibiting metastasis. Therefore, the conjugation of prosenescent cancer therapies and senolytic drugs offers the best outcome [159,160,161]. One method entails encapsulating senolytics in nanomaterials capable of identifying and inhibiting senescent cells, consequently reversing senescence induced on cancer patients from previous chemotherapeutic drugs or radiation. Conducting senescent mitigation this way can occur by either directly destroying the cells or opting to temper the senescent secretion signaling pathways inflicting the damage [162].

A study deploying tannic acid-docetaxel self-assemblies (DSAs) following prostate cancer chemotherapy with docetaxel is a prime example of nanotherapeutics used for antisenescent targeting [163]. The DSA exposure enacted TGFβR1/Forkhead box protein O1 (FOXO1)/p21 signaling intervention in vitro on docetaxel-treated biological assays, as well as the activation of apoptosis in senescent cells. An additional study observing mice treated with the chemotherapeutic drug Palbociclib exemplifies the advantageous nature of nanotherapy with galacto-oligosaccharide-encapsulating cytotoxic drugs that target the drug-induced senescent cells [164]. The gal encapsulation enables the preferential release of the internal cargo when exposed to high lysosomal β-galactosidase levels—a key biomarker to senescent cells. What the specificity in treatments such as these offers is lowered side effects upon the systemic dissemination of the senolytic drugs, as well as diminished toxicity of the chemotherapeutics used. Overall, senotherapy amidst or following chemotherapy or radiation presents tremendous value in helping reverse the senescence-evoked contributions to cancer relapse. In Table 2, we listed the senotherapies used in clinical trials or experimental settings.

**Table 2:** Senotherapeutic drugs targeting senescent cells or SASPs in different age-related diseases.

Senotherapeutic Drugs	Class	Targeted Diseases	Status
Dasatinib+Quercetin	Senolytic	Alzheimer disease, aging	Clinical trial: NCT04063124, NCT05422885
Quercetin	Senolytic, senomorphic	Coronary artery disease	Clinical trial: NCT04907253
Navitovlax	Senolytic	Clearing senescent bone marrow hematopoietic stem cells (HSCs) and senescent muscle stem cells (MuSCs) from aged mice or mice under irradiation	[165]
		Clearing senescent osteoarthritic chondrocytes in osteoarthritis	[166]
Cardiac Glycosides (Ouabain, Digoxin, and Proscillaridin A)	Senolytic	Lung fibrosis, elimination of apoptotic cells	[167]
Fisetin	Senolytic, senomorphic	Aging, progeroid mice model	[168]
UBX0101	Senolytic	Osteoarthritis, knee, treating degenerative joint disease	Clinical trial: NCT03513016 [169,170]
UBX1967	Senolytic	Pathological neovascularization (NV)	https://iovs.arvojournals.org/article.aspx?articleid=2774894 (accessed on 28 January 2023)
UBX1325	Senolytic	Neovascular age-related macular degeneration	NCT04537884, NCT05275205
Curcumin	Senolytic	Cardiovascular risk factor, vascular aging, aging	NCT04119752, NCT01968564 [171]
Curcumin Analog EF24	Senolytic	Senolytic elimination of senescent endothelial cells, senescent fibroblast	[172]
A1331852	Senolytic	Eliminate senescent cells (HUVEC) and IMR90	[173]
A1155463	Senolytic	Eliminate senescent cells (HUVEC) and IMR90	[173]
Hsp90 inhibitors (Geldanamycin, Tanespimycin, Alvespimycin)	Senolytic	Elimination of senescent cells in vitro	[174]

## 6. Conclusions

The widespread lymphatic remodeling enacted by the SASP of senescent cells exacerbates cancer metastasis and should be studied and used in treatment to prevent relapse in patients. The prosenescent mechanisms inherent throughout most of the cancer treatments may initially obstruct tumor cell proliferation; however, the ensuing inflammation and enhanced lymphangiogenesis as a result of SASP factors such as IL-6, IL-8, VEGFs, and more threatens a broadened invasion through higher cancer cell uptake. Consequently, the pairing of these treatments with additional senolytic drugs equipped to modulate senescent cell accumulation offers a solution for the duality senescence posed to a tumor environment. The present research displays the active role that senescent cells acquire to restructure the tumorigenic microenvironments proximal to lymphatic vasculatures into skilled networks for cancer cell permittance and transport, all of which sustain the emergence of the lymphatic system as a notable contributor to cancer metastasis. However, the detailed mechanisms are grossly understudied. Further research studying senotherapy used in conjugation with prosenescent chemotherapy or radiation should be conducted with a focus on lymphatic pathophysiology. This allows for the specific monitoring of lymphatic remodeling associated with senescence—a phenomenon still not widely studied—as well as insight into how senolytic drugs could potentially regulate this occurrence. With these future efforts, understanding the dichotomy of prosenescent benefits and consequences on the overall lymphatic augmentation of metastasis can aid in improving the treatment plans for cancer patients, and thus demands to be explored.

## Figures and Tables

**Table 1:** List of anticancer therapies that induce senescence.

Class	Name of the Drug	Cancer Types	Reference
Chemotherapy	Doxorubicin	Cervical cancer (HeLa cells), hepatocellular carcinoma (HuH7), colorectal carcinoma, breast cancer	[34,43]
	Etoposide	Adrenocortical H295R cells, epithelial carcinoma (A549), adrenocortical tumor cells	[44,45]
	Bleomycin	Pulmonary fibrosis, alveolar epithelial cells	[46]
	Cisplatin	Ovarian cancer, nasopharyngeal carcinoma cells, lung cancer	[40,47]
	Mitoxantrone	Dermal fibroblasts, prostate cancer	[48,49]
	Temozolomide	Glioma, melanoma	[50,51]
	Paclitaxel	Non-small-cell lung cancer cells, breast cancer	[52,53]
	Methotrexate	Breast cancer, colon cancer, adenocarcinoma	[53,54]
	Camptothecin	Colorectal cancer	[55]
Radiation therapy		Breast cancer, glioblastoma, non-small-cell lung cancer	[56,57]
Immune therapy	Rituximab	B-cell lymphoma	[58,59]

## Data Availability

Not applicable.
